# Mental health problems among adolescents and young adults with childhood-onset physical disabilities: A scoping review

**DOI:** 10.3389/fresc.2022.904586

**Published:** 2022-09-06

**Authors:** Shalini Lal, Stephanie Tremblay, Danielle Starcevic, Melina Mauger-Lavigne, Dana Anaby

**Affiliations:** ^1^ School of Rehabilitation, Faculty of Medicine, University of Montreal, Montreal, QC, Canada; ^2^ Health Innovation and Evaluation Hub, University of Montreal Hospital Research Centre, Montreal, QC, Canada; ^3^ PEPP Montreal and ACCESS Open Minds, Douglas Mental Health University Institute, Montreal, QC, Canada; ^4^ School of Physical and Occupational Therapy, McGill University, Montreal, QC, Canada; ^5^ Centre for Interdisciplinary Research in Rehabilitation of Greater Montreal (CRIR), Montreal, QC, Canada

**Keywords:** mental health, youth, mental health services, access, disability, systematic review, early intervention, psychiatry

## Abstract

**Aim:**

This scoping review aims to better understand the extent and nature of research activity on the topic of mental health problems in young people with childhood-onset physical disabilities. Specifically, we document what has been investigated in terms of the occurrence and experience of mental health problems among young people with childhood-onset physical disabilities, and their access to mental health services.

**Methods:**

We searched four databases (Medline, PsycINFO, CINAHL, Embase) for articles published between 2007 and 2019. Studies were included if they addressed: (1) young people between the ages of 13 and 24 with a childhood-onset physical disability, and (2) mental health assessment, treatment, or service access and use.

**Results:**

We identified 33 peer-reviewed studies that focused mainly on young people with cerebral palsy, juvenile arthritis, and spina bifida. The most common mental health problems investigated were depression and mood related difficulties (73%), anxiety (39%), and social/behavioural issues (33%) and the most common age range was 13 to 17. Ten studies explored access, use, and experiences of mental health services; stigma; caregiver mental health; and value for comprehensive care, using qualitative, quantitative, or mixed methods.

**Conclusions:**

Findings suggest the importance of developing integrated models of service delivery to identify and address the mental health needs of this population, and consensus on best practices for assessment and reporting rates of subclinical symptoms and psychiatric conditions.

## What this paper adds:

• Majority of studies pertained to: cerebral palsy, juvenile arthritis, and spina bifida.
• Most prevalent mental health concerns investigated: depression/mood, anxiety, social/behavioural difficulties.
• Large amount of variation in reported rates of mental health problems.
• Methodological gaps including inconsistencies in mental health assessments used.
• Need for integrated models of service delivery to increase access to peer-to-peer and vocational support services to support youth with physical disabilities in their mental health and well-being.

## 1. Introduction

Childhood-onset physical disabilities such as cerebral palsy, spina bifida, and juvenile arthritis are often associated with restrictions in mobility, self-care, leisure, and community activities (1–3), which can negatively impact mental health and well-being. Indeed, research has shown that physical disabilities in young people have been associated with subclinical mental health problems, including depressive and anxious symptoms (4–6). This is concurrent with epidemiological research demonstrating that, compared to individuals of other age groups, young people in general between the ages of 12–24 have a higher risk of developing a mental illness (7). Having a physical disability during adolescence and young adulthood compounds this risk given that it is a developmental stage when comparisons with peers are common, and awareness of these differences (e.g., reduced mobility) can affect self-esteem (8–10). Students with physical disabilities are twice as likely to report feeling sad or hopeless on a daily basis and three times as likely to report attempting suicide compared to their typically developing peers (11).

Mental health problems and psychiatric conditions in young people with childhood-onset physical disabilities are concerning as these, if left untreated, can often persist or worsen throughout adulthood (12). For this population, adolescence and young adulthood are phases of life characterized by a range of developmental transitions (8) that are coupled with a shift from pediatric to adult care services which is often accompanied by a reduction of health-related services. Additionally, young people with childhood-onset physical disabilities typically receive services in rehabilitation settings with therapists specialized in physical rehabilitation who may have limited awareness and skills needed to identify, assess, and address mental health issues in their clientele.

Thus, understanding the mental health and related needs of adolescents and young adults with physical disabilities and their access and use of mental health services is necessary for identification, prevention and early intervention. Such knowledge can also inform the development of integrated rehabilitation services that can comprehensively address the mental health and well-being of young people with physical disabilities. However, limited efforts have been made to synthesize knowledge about the occurrence of mental health problems in this population and their experiences of accessing and using mental health services.

### 1.1. Objective

The purpose of this scoping review is to map and synthesize research on mental health problems among young people with congenital and childhood-onset physical disabilities associated with restricted mobility, and their access and use of mental health services.

## 2. Materials and methods

We used scoping review methodology to address the objectives of this study. Scoping reviews focus on broader topics (e.g., subclinical symptoms versus a specific mental disorder) and may include a multitude of study designs (13). There are four primary reasons why one might complete a scoping review: 1) to map the extent and nature of research activity on a topic; 2) to determine whether a full systematic review can/should be completed; 3) to synthesize and disseminate findings from research; and 4) to identify gaps in the literature (13). In line with reasons 1, 3, and 4, the current scoping review aims to obtain a portrait of the emerging literature in order to identify the extent and nature of the research, synthesize and disseminate findings related to objectives, and identify research gaps.

We developed a scoping review protocol based on the framework proposed by Arksey and O'Malley (13) and additional methodological resources including Levac et al. (14), the Joanna Briggs Institute (15), and the PRISMA-ScR Checklist (16). The protocol was registered online with Open Sciences Framework (17). We also examined the PRISMA-ScR Checklist in relation to the current report to ensure alignment with the latest scoping review reporting guidelines (see **Supplementary File Table 1** for the completed PRISMA-ScR Checklist).

### 2.1. Identifying the research question

Using the PCC framework (Population/Participants, Concept, Context), this review aimed to answer the following research question: In young people recruited from community and clinical settings between the ages of 13 to 24 with congenital and childhood-onset physical disabilities (e.g., cerebral palsy, spina bifida, muscular dystrophies, juvenile idiopathic arthritis, or other conditions that affect movement and mobility), what is known about the occurrence of mental health problems, their experience of these problems, and their access and use of mental health services?

### 2.2. Identifying relevant studies

#### 2.2.1 Information sources

The search was focused on peer-reviewed, scientific publications. We conducted the search in four electronic databases (Medline, Embase, PsycINFO and CINAHL) that cover a range of research areas including health, psychology, and rehabilitation sciences. In consultation with a university librarian specializing in health and rehabilitation, we developed an initial search strategy for Medline (see Table 1) and adapted the strategy for the other databases. The keyword strategy included the following concepts: physical disability or diagnosis of congenital or childhood-onset physical disability AND mental health diagnosis or symptoms AND adolescents (13 to 18) or young adults (19 to 24). The upper limit for age was set to 24 years old which corresponds with the United Nations Educational, Scientific and Cultural Organization (UNESCO) definition of youth. The keywords for capturing the construct of physical disability (further detailed below) were based on a previous scoping review (18). The results in Medline were restricted to humans, French and English languages, and years of publication from 2007 onwards. Language restrictions were based on the languages the authors are fluent in, the lower limit for the publication year was selected to restrict the number of results (for feasibility) and ensure the historical relevance of information, especially as it pertains to access and use of mental health services. The search was initially implemented December 2016, however given that a small number of papers were found, we were unable to justify a systematic review that addressed the research questions. Therefore, we updated our search strategy in May 2019.

**Table 1:** Medline search strategy.

**Physical disability** Search field (Title, abstract, keyword) (Physical* disab* or Physical* handicap* or Physically challenged or Physically disabled or Cerebral Palsy or Spina Bifida or Myelomeningocele or Meningocele or Spinal muscular Atrophy or Duchenne Muscular Dystrophy or Congenital Deformit* or Juvenile Arthr*).ab,kf,kw,ti. Advanced search exp cerebral palsy/ or exp spinal Dysraphism/ or exp Meningomyelocele/ or exp meningocele/ or exp muscular atrophy, spinal/ or exp Muscular Dystrophy, Duchenne/
AND
**Mental health disorder** Search field (Title, abstract, keyword) (Mental* ill* person* or Mental health* or Psychiatric diagnosis or Psychotic disorder* or Brief reactive psychosis or Schizoaffective disorder* or Schizophreniform disorder* or Psychosis or Schizophrenia or Schizophrenic disorder* or Bipolar disorder* or Bipolar depression or Manic disorder* or Manic state* or Mania or Bipolar affective psychosis or Anxiety or Depression or Substance* abuse* or Substance* related disorder* or Eating disorder* or ASD or Autism or DSM).ab,kf,kw,ti. Advanced search mental disorders/ or exp anxiety disorders/ or exp “bipolar and related disorders"/ or exp “disruptive, impulse control, and conduct disorders"/ or exp dissociative disorders/ or exp elimination disorders/ or exp “feeding and eating disorders"/ or exp mood disorders/ or exp neurocognitive disorders/ or exp anxiety, separation/ or exp “attention deficit and disruptive behavior disorders"/ or exp child behavior disorders/ or exp child development disorders, pervasive/ or exp intellectual disability/ or exp learning disorders/ or exp reactive attachment disorder/ or exp schizophrenia, childhood/ or exp paraphilic disorders/ or exp personality disorders/ or exp “schizophrenia spectrum and other psychotic disorders"/ or exp sexual dysfunctions, psychological/ or exp somatoform disorders/ or exp substance-related disorders/ or exp “trauma and stressor related disorders"/
AND
**Young adults and adolescents** Search field (Title, abstract, keyword) (Youth* or Adolescen* or Young adult* or teen or teens or teenager*).ab,kf,kw,ti. Advanced search exp adolescent/ or exp young adult/

### 2.3. Selecting studies

#### 2.3.1. Selection of sources of evidence

After the search was implemented in all four databases, the results were imported into Endnote X7. Duplicates were identified and removed. First-level screening (based on title and abstract) was completed using the inclusion and exclusion criteria. Studies that did not meet these criteria were removed (first-level screening was split between two reviewers, A2 & A4). Four reviewers then completed second level (full text) screening of the remaining articles using the inclusion and exclusion criteria (A1, A2, A4, A5). Disagreements were resolved through discussion between research team members. Articles that met the eligibility criteria after second level (full text) screening underwent data extraction using Excel software.

#### 2.3.2. Inclusion and exclusion criteria

Cerebral palsy (CP), spina bifida (SB), muscular dystrophies, juvenile idiopathic arthritis (JIA), or other conditions that affect movement and mobility (e.g., Mobius disorder) were the focus of this review given their associated physical disabilities. Cerebral palsy (CP) defines a group of non-progressive neurodevelopmental disorders that affects posture and movement, and individuals can also experience comorbid sensation, perception, cognition, communication, behavior, musculoskeletal impairments and/or epilepsy (19). Spina bifida is a health problem caused by a congenital deformity of the neural tube, and is associated with deformities, impaired sensation, muscle weakness, paralysis, orthopedic problems, bladder and bowel dysfunction and seizure disorders (20). Muscular dystrophy is a group of disorders (the most common being Duchenne's) caused by a genetic irregularity resulting in muscle weakness, hypotonia and atrophy (9). Juvenile idiopathic arthritis (JIA) is an umbrella term for arthritic illnesses that begin before the age of 16 and involve inflammation of one or more joints, alongside other symptoms depending on the type of JIA (21). Other physical disabilities that affect movement and mobility include disorders such as Mobius disorder, a congenital disorder causing partial or complete paralysis of facial nerves, often in conjunction with malformation of upper and/or lower limbs (22).

The following mental health symptoms and conditions were included: psychotic disorder, brief reactive psychosis, schizoaffective disorder, schizophreniform disorder, psychosis, schizophrenia, bipolar disorder, bipolar depression, manic disorder, manic state, mania, bipolar affective psychosis, anxiety, depression, substance abuse, substance-related disorder, eating disorder, and autism spectrum disorder (ASD) or autism, as per the *Diagnostic and Statistical Manual of Mental Disorders, 5th edition* (DSM-V) (23). In addition, studies were included if they focused on behavioral difficulties, such as hyperactivity, aggressivity, and emotional lability. Studies that assessed general psychiatric symptoms were also included, given that the first symptoms or experiences of illness tend to present themselves around adolescence before a formal diagnosis has been given.

The inclusion criteria for the selection of articles were as follows: (1) study population with a mean age between 13 and 24 years old, or at least 50% of the studied sample between ages 13 and 24 with specific results reported about this group, or including the term *adolescents, youth,* or *young adults* to describe the population if no age is mentioned; (2) study population including at least 50% of individuals diagnosed with a disability noted above (e.g., cerebral palsy, spina bifida), or mental health data for this population were reported separately; (3) study addressing psychological issues, mental illnesses or symptoms of mental illness; (4) article published in French or English, between 2007 and 2019. In terms of exclusion criteria, literature reviews and grey literature were excluded (reference lists of literature reviews were scanned).

### 2.4. Charting the data

#### 2.4.1. Data charting process

Using Excel software, a data charting form was developed by adapting a template used in A1′s previous scoping reviews. The research team piloted and finalized the chart at the start of the data extraction process. Articles that were selected for inclusion in the study were read in full to establish familiarity with the data. Data extraction was first conducted by one reviewer (A2 or A4) and audited by another reviewer to confirm the completeness and accuracy of the extracted data (A2 or A3). The data extraction was also audited by A1 and A5 at several stages throughout the process.

#### 2.4.2. Data items

Data selected for extraction included: study characteristics (author, year, title, country), study objective(s), study population and research design, methods and measures, and results. Only data related to the main objectives of the scoping review were extracted (e.g., only measures, methods, and results pertaining to mental health or access or use of mental health services).

#### 2.4.3. Critical appraisal of individual sources of evidence

A critical appraisal of the sources of evidence is an optional step in the conduct of scoping reviews (18). We did not conduct this step due to the large variability of study designs and research approaches within the selected studies and given our primary objective of mapping the evidence.

### 2.5. Collating, summarizing, and reporting the results

#### 2.5.1. Characteristics of sources of evidence

Counts, proportions, and tables were used to synthesize study characteristics such as country, study design, year, study population characteristics (e.g., type of diagnosis, age), and measures used to assess mental health.

#### 2.5.2. Occurrence of mental health problems

A list of the mental health problems investigated in the selected studies was created and two reviewers (A2, A3) separately counted the number of studies that investigated each mental health problem. The final counts were discussed between the two reviewers, with a third reviewer (A1) resolving any disagreements.

The prevalence rates of mental health problems within the study populations were summarized using frequencies, proportions, means, medians, and ranges. A prevalence rate was defined as the number of participants identified as having a case of a mental health problem divided by the total number of participants in the study (reported either directly by the authors in text or in a table within the selected study or calculated manually by the current scoping review authors). A “case of a mental health problem” was counted if either (a) the authors of the studies identified the concern as a “clinical case” based on the measures and thresholds they used to evaluate mental health, including mild, moderate, or severe mental health concerns, or (b) if a participant (the young person themselves or a parent/guardian) reported, in a self-report measure or clinical interview, that they had or perceived having a mental health problem. If authors reported on the number of “borderline” or “at risk” cases (i.e., scores or symptoms that do not meet clinical thresholds but are beyond the normal range) of a mental health disorder, these were not considered in the current summary of prevalence rates, with the exception of studies that were categorized as “mental health not specified” – given that this is a broader concept. For the rest of the categories, we did not include “borderline” or “at risk” as these items were not consistently defined nor reported across the selected studies. We included self-reported cases given that these reports were used as the only measures of mental health within several of the selected studies. One reviewer (A3) counted the number of studies that reported rates of a problem within each mental health problem category, extracted the rates from the studies, and calculated the mean, median, and range of the rates. A second reviewer (A1) validated the extraction and calculation of rates and any issues identified were resolved by discussion between the two reviewers.

#### 2.5.3. Experience of mental health problems, access and use of mental health services

An inductive content analysis (24) was used to synthesize extracted findings related to access and use of mental health services among the study population. First, the 33 selected studies were reviewed and articles that included qualitative data on access to mental health services among young people with childhood-onset physical disabilities were identified (10 articles based on either a mixed methods design or solely a qualitative methodology). Two reviewers (A2, A3) then separately extracted results from these ten articles and separately coded the results to determine a preliminary list of themes. The final list of themes and subthemes was determined by discussion and consensus between the two reviewers in consultation with a third reviewer (A1). Themes were defined as any thematically-related results that appeared in at least 2 of the studies; subthemes were defined as any thematically-related results that appeared in at least 2 of the studies within the identified theme that were distinct from the other results. Counts, proportions, and tables were used to provide a final summary of these findings.

## 3. Results

**Figure 1:**
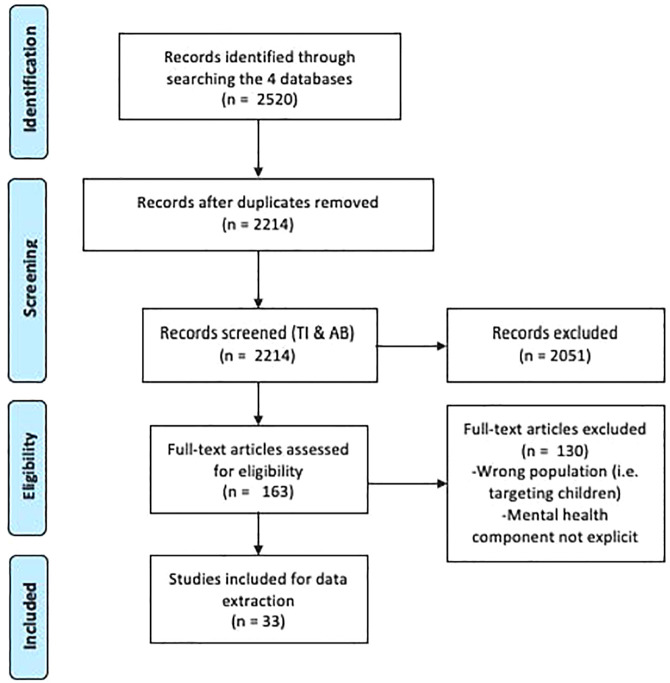
PRISMA flow diagram.

A total of 2,520 articles were retrieved and imported into Endnote X7, and 2,214 remained after removing duplicates. After first-level screening, 2051 articles were excluded because they did not meet the inclusion and exclusion criteria. Next, 163 articles underwent second-level screening, and 33 documents were included in the current scoping review. The 130 articles that were excluded during the second-level screening were either targeting the wrong population, did not explicitly address a mental health component, or did not include sufficient detail for data extraction. See Figure 1 for an adapted PRISMA flow diagram (25).

### 3.1. Overview of studies reviewed

#### 3.1.1. Characteristics of sources of evidence

**Figure 2:**
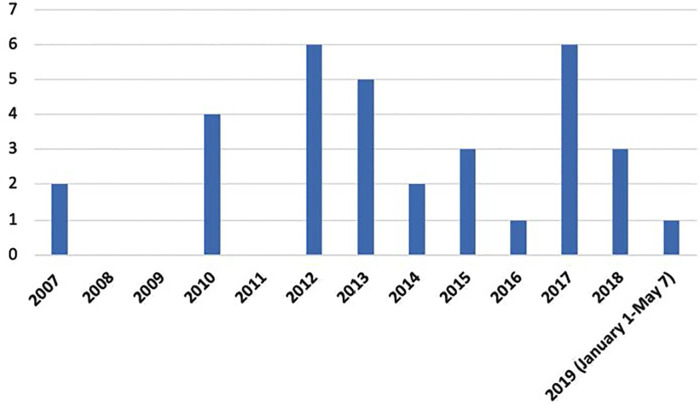
Number of articles published per year.

**Supplementary File Table 2** provides an overview of the 33 studies reviewed (4, 26–57). Articles originated from several different countries, with the largest proportion (13/33) published in the United States (see Table 2). In terms of research design, there were 21 cross-sectional studies, 5 longitudinal studies, 3 case studies, 3 qualitative studies, and 1 retrospective cohort study. All the articles were published between 2007 and 2019, with most appearing after 2010 (see Figure 2). The largest portion of articles focused on cerebral palsy (*n* = 12), followed by juvenile idiopathic arthritis (*n* = 8) and spina bifida (*n* = 8). The other physical disabilities were muscular dystrophies (*n* = 3) and Mobius syndrome (*n* = 1), and one article included several types of disabilities.

**Table 2:** Number of articles per country.

Country	Number (*n *= 33)
United States	13
United Kingdom	4
Canada	2
Italy	2
Norway	2
Australia	1
Colombia	1
Denmark	1
Egypt	1
Germany	1
Greece	1
Netherlands	1
Serbia	1
Taiwan	1
Europe	1

#### 3.1.2. Methods used to capture mental health

As illustrated in **Supplementary File Table 3**, we identified 36 methods (majority being measures) used among the 33 studies to assess mental health symptoms or related phenomena such as quality of life and pain. The most common used scales across studies were: Children's Depression Inventory (CDI; *n* = 7), Strengths and Difficulties Questionnaire (SDQ-25; *n* = 6), Child Behavior Checklist (CBCL; *n* = 5), Pediatric Quality of Life Inventory (PedsQL; *n* = 3), Wechsler Intelligence Scale (*n* = 3), Child Attitude Towards Illness Scale (CATIS; *n* = 2), Child Health Questionnaire (CHQ; *n* = 2), Mental Status Examination (*n* = 2) and the Mood and Feelings Questionnaire (MFQ; *n* = 2). The remaining measures (*n* = 27) were used in only one study each. Measures were a mix of young people and parent reports, and two measures (the General Health Questionnaire and the Social Orientation of Parents with Handicapped Children Questionnaire) served to capture parents' mental health states. Interviews of various types (e.g., open-ended, psychiatric, semi-structured, or structured) were also commonly used (*n* = 10).

#### 3.1.3. Prevalence of mental health problems and cognitive, physical and functional difficulties among young people with childhood-onset physical disabilities

The most common mental health problems addressed in the studies were depression and mood related difficulties (*n* = 24), anxiety (*n* = 13), and social/behavioural difficulties (*n* = 11). Table 3 synthesizes the mental health problems reported in the studies, and is organized in relation to physical health condition. Table 4 provides a synthesis of the reported rates for each mental health concern, including the number of studies that reported the rates, and the range, mean, and median of the rates. The number of articles reporting rates were: 12 out of 24 for depression and mood related difficulties, 8 out of 13 for anxiety, 5 out of 11 for social/behavioural difficulties, 6 out of 9 for mental health problems not specified, including aggregated rates of mental health problems, 3 out of 6 for attention deficit hyperactivity disorders, 2 out of 3 for autism spectrum disorders, 0 out of 2 for psychosis, 0 out of 1 for self-harm and 1 out of 1 for substance use behaviors. The presence of mental health symptoms found in these studies are further detailed in the sections below. All rates are summarized to 1 decimal point.

**Table 3:** Number of articles addressing a specific mental health problem (*n* = 33)^a^.

Mental Health Problem	Number (%)	Studies
Depression and Mood Related Difficulties	25 (76%)	***Spina Bifida***
		3. Bellin et al., 2010
		7. Essner & Holmbeck, 2010
		13. Kelly et al., 2012
		17. Nicholls et al., 2015
		25. Soe et al., 2012
		28. Verhoef et al., 2007
		30. Wagner et al., 2015
		***Juvenile Arthritis***
		1. Abdul-Sattar et al., 2014
		11. Hanns et al., 2018
		12. Hanson et al., 2018
		18. Ramsey et al., 2013
		22. Russo et al., 2012
		26. Stevanovic & Susic, 2013
		27. Tong et al., 2013
		29. Wagner et al., 2007
		***Muscular Dystrophy***
		6. Colombo et al., 2017
		14. Latimer et al., 2017
		15. Lindsay et al., 2017
		***Cerebral Palsy***
		9. Foster et al., 2010
		20. Ramstad et al., 2015
		21. Rapp et al., 2017
		23. Sienko, 2018
		31. Whitney et al., 2019
		***Other***
		4. Briegel et al., 2010
Anxiety	13 (39%)	***Spina Bifida***
		3. Bellin et al., 2010
		13. Kelly et al., 2012
		17. Nicholls et al., 2015
		***Juvenile Arthritis***
		12. Hanson et al., 2018
		22. Russo et al., 2012
		26. Stevanovic & Susic, 2013
		***Muscular Dystrophy***
		6. Colombo et al., 2017
		14. Latimer et al., 2017
		***Cerebral Palsy***
		8. Florou et al., 2016
		10. Grody & Coffey, 2012
		31. Whitney et al., 2019
		***Other***
		4. Briegel et al., 2010
		33. Yang et al., 2017
Social/Behavioural Difficulties	11 (33%)	***Juvenile Arthritis***
		22. Russo et al., 2012
		27. Tong et al., 2013
		***Muscular Dystrophy***
		15. Lindsay et al., 2017
		***Cerebral Palsy***
		2. Adegboye et al., 2017
		5. Brossard-Racine et al., 2013
		8. Florou et al., 2016
		10. Grody & Coffey, 2012
		31. Whitney et al., 2019
		20. Ramstad et al., 2015
		***Other***
		4. Briegel et al., 2010
		33. Yang et al., 2017
Mental Health Problems (not specified, includes aggregated rates of mental health problems)	9 (27%)	***Spina Bifida***
		3. Bellin et al., 2010
		28. Verhoef et al., 2007
		30. Wagner et al., 2015
		***Juvenile Arthritis***
		22. Russo et al., 2012
		***Cerebral Palsy***
		19. Ramstad et al., 2012
		20. Ramstad et al., 2015
		***Other***
		4. Briegel et al., 2010
		32. Woodward et al., 2012
		33. Yang et al., 2017
Attention Deficit Hyperactivity Disorders	6 (18%)	***Muscular Dystrophy***
		14. Latimer et al., 2017
		***Cerebral Palsy***
		2. Adegboye et al., 2017
		5. Brossard-Racine et al., 2013
		20. Ramstad et al., 2015
		31. Whitney et al., 2019
		***Other***
		33. Yang et al., 2017
Autism Spectrum Disorders	3 (9%)	***Muscular Dystrophy***
		6. Colombo et al., 2017
		***Cerebral Palsy***
		16. Mouridsen et al., 2013
		***Other***
		33. Yang et al., 2017
Psychosis	2 (6%)	***Cerebral Palsy***
		9. Foster et al., 2010
		10. Grody & Coffey, 2012
Self-harm	1 (3%)	***Spina Bifida***
		24. Singhal et al., 2014
Substance Use and Other Health Risk Behaviours	1 (3%)	***Spina Bifida***
		25. Soe et al., 2012

^a^ For each study cited within this table, the first number in the third column represents the selected studies presented in alphabetical order in **Supplementary File Table 2**: Overview of Studies Reviewed (1–33).

**Table 4:** Studies reporting rates of mental health problems.

Mental Health Problem	Number of Studies (out of total number of studies pertaining to mental health problems)^a^	Measure Used to Determine Rates	Rates (Range, Mean, Median)
Depression and Mood Related Difficulties^b^	12/25 1. Abdul-Sattar et al., 2014 3. Bellin et al., 2010 4. Briegel et al., 2010 6. Colombo et al., 2017 11. Hanns et al. 2018 14. Latimer et al., 2017^c^ 17. Nicholls et al., 2015 22. Russo et al., 2012^d^ 23. Sienko, 2018 25. Soe et al., 2012 30. Wagner et al., 2015 31. Whitney et al., 2019	Children's Depression Inventory (CDI) – 3 studies Hopkins Symptom Checklist (HSCL-25) Child Behaviour Checklist (CBCL) – 3 studies Mood and Feelings Questionnaire (MFQ) Scale developed by investigators (i.e., Family Quality of Life survey - reported by caregivers, Spina Bifida Secondary Conditions (SBSC) survey) Patient Health Questionnaire-9 (PHQ-9) Centre for Epidemiological Studies Depression Scale (CES-D) 2016 National Survey of Children's Health (reported by parents of child) Psychiatric Diagnosis	Range: 7.8% to 53.0% Mean: 26.5% Median: 22.2%
Anxiety^e^	8/13 3. Bellin et al., 2010 4. Briegel et al., 2010 6. Colombo et al., 2017 14. Latimer et al., 2017^c^ 17. Nicholls et al., 2015^d^ 22. Russo et al., 2012 31. Whitney et al., 2019 33. Yang et al., 2017	Hopkin's Symptom Checklist (HSCL-25) Child Behaviour Checklist (CBCL) – 2 studies Scale developed by investigators (i.e., Family Quality of Life survey - reported by caregivers) Revised Children's Manifest Anxiety Scale (RCMAS-2) 2016 National Survey of Children's Health (reported by parents of child) Psychiatric Diagnosis Mini International Neuropsychiatric Interview for Children and Adolescents (MINI-KID) Anxiety Checklist for Children and Adolescents	Range: 2.2% to 31.0% Mean: 15.9% Median: 14.4%
Social/Behavioural Difficulties	5/11 4. Briegel et al., 2010 5. Brossard-Racine et al., 2013 22. Russo et al., 2012 31. Whitney et al., 2019 33. Yang et al., 2017	Child Behaviour Checklist (CBCL) - 2 studies (one study used the Chinese version) Social Difficulties Questionnaire (SDQ) 2016 National Survey of Children's Health (reported by parents of child) Psychiatric Diagnosis	Range: 16.0% to 27.3% Mean: 20.9% Median: 18.8%
Mental Health Problems (Not specified, includes aggregated rates of Mental Health problems)^g^	6/9 3. Bellin et al., 2010 4. Briegel et al., 2010^f^ 20. Ramstad et al., 2015 22. Russo et al., 2012 30. Wagner et al., 2015 33. Yang et al., 2017	Strengths and Difficulties Questionnaire (SDQ-25; Norwegian version) Open-ended clinical interviews Scale developed by investigators (i.e., Spina Bifida Secondary Conditions (SBSC)) Child Behaviour Checklist (CBCL) – 2 studies (one study used Chinese version) Hopkins Symptom Checklist (HSCL-25) Mini International Neuropsychiatric Interview for Children and Adolescents (MINI-KID)	Range: 16.0% to 69.0% Mean: 43.1% Median: 45.4%
Attention Deficit Hyperactivity Disorders	3/6 14. Latimer et al., 2017^c^ 31. Whitney et al., 2019 33. Yang et al., 2017	Scale developed by Investigators (i.e., Family Quality of Life Survey - reported by caregivers) 2016 National Survey of Children's Health (reported by parents of child) Psychiatric Diagnosis Mini International Neuropsychiatric Interview for Children and Adolescents (MINI-KID)	Range: 16.7% to 19.7% Mean: 18.6% Median: 19.5%
Autism Spectrum Disorders^h^	2/3 6. Colombo et al., 2017 33. Yang et al., 2017	Autism Diagnostic Observation Schedule (ADOS) Combined information from diagnostic tools, observations of the subject, and parental report	Range: 3.3% to 14.9% Mean: 9.1% Median: 9.1%
Psychosis	0/2	N/A	N/A
Self-harm^i^	0/1	N/A	N/A
Substance Use and Other Health Risk Behaviors	1/1 25. Soe et al., 2012^j^	Interviews Health related questionnaires	Range: 11.0-92.0%^j^

^a^ For each study cited within this table, the numbers in the second column represent the selected studies presented in alphabetical order in **Supplementary File Table 2**: Overview of Studies Reviewed (1-33).

^b^ The anxious/depressed and withdrawn/depressed scales on the CBCL were used as an estimate measure of depression.

^c^ Includes combined prevalence rates from participants with both Duchenne and Becker Muscular Dystrophy.

^d^ Russo et al. included prevalence rates of depression and anxiety using different assessment methods; Nicholls et al. reported separate rates for two components of anxiety. In cases where two separate rates were provided for the same mental health issue, the rates were averaged before using them to calculate the overall summary mean, median and range estimates.

^e^ The anxious/depressed scale on the CBCL was used as an estimate measure of anxiety.

^f^ The prevalence estimate includes participants outside of our target age group.

^g^ Borderline cases were included in the calculations exceptionally for this broad category.

^h^ One study that assessed Autism Spectrum Disorders had a sample of participants with Autism Spectrum Disorder and reported the prevalence rate of Cerebral Palsy within the sample. This rate was not included in the calculation of mean, median, range.

^i^ The study reported that 34 individuals between the ages of 10–24 with Spina Bifida had an incidence of self-harm, however the total number of individuals between the ages of 10–24 with Spina Bifida included in the study was not reported. Therefore, the rate of self-harm in this population could not be calculated.

^j^ The study reported prevalence rates of a range of health risk behaviors (e.g., binge drinking, lack of exercise), given the conceptual differences between the health risk behaviors reported, only the range is reported.

##### 3.1.3.1. Depression and mood related (*n* = 25)

Among the 25 studies that addressed depression and mood-related difficulties, seven were focused on spina bifida (27, 30, 33, 36, 42, 45, 47), eight on juvenile arthritis (4, 26, 32, 37, 40, 43, 44, 46), three on muscular dystrophy (29, 34, 35), five on cerebral palsy (31, 38, 39, 41, 48, 49), and one on other conditions (28). Twelve of these studies reported on rates of depression and mood-related difficulties, which ranged from 7.8% to 53.0%. Specifically, depression and mood-related symptoms in participants with spina bifida, ranged from 13.6% to 53.0% (27, 36, 42, 47); juvenile arthritis, 14.7% to 36.2% (4, 26, 40); muscular dystrophy, 8.5% to 27.6% (29, 34); cerebral palsy, 7.8% to 42.0% (41, 48), and other conditions, 8.3% (28). When comparing the presence of depression and mood-related symptoms in youth with physical disabilities to that of typically developing youth, results were inconsistent across and even within studies, depending on the assessment method. For example, some studies found that youth with physical disabilities had greater levels of mood or depressive symptoms (e.g., [36, 38, 42]), whereas other studies found no differences among the groups in emotional and psychological health (e.g., [40, 45, 48]). An example of within study inconsistency is Russo et al. (40) which found significant differences in the presence of depression or mood related symptoms when using standardized methodology (i.e., no psychopathology found) versus using open ended interviews and clinical observation in a sample of youth with JIA (i.e., 33% presenting with emotional lability).

##### 3.1.3.2. Anxiety (*n* = 13)

Among the 13 studies that addressed anxiety, three were focused on spina bifida (27, 33, 36) and juvenile arthritis (32, 40, 43), two on muscular dystrophy (29, 34), three on cerebral palsy (48, 49, 50), and two on other conditions (28, 51). Among these 13 studies, 8 reported on rates of anxiety, specifically in relation to spina bifida (27, 36), juvenile arthritis (40), muscular dystrophy (29, 34), cerebral palsy (48), and other conditions (28, 51). Across these conditions, anxiety symptoms were reported to be present in 2.2% to 31.1% of the participants. Specifically, anxiety symptoms in participants with spina bifida, ranged from 20.5% to 31.1% (27, 33, 36); juvenile arthritis, 4% (40); muscular dystrophy, 2.2% to 27.6% (29, 34); cerebral palsy, 30.2% (48), and other conditions, 3.3% to 8.3% (28, 51). When studies compared rates of anxiety in youth with physical disabilities to typically developing youth, results were inconsistent, such that some found youth with physical disabilities had greater levels of anxiety symptoms (e.g., [33, 48]), whereas others found no differences (e.g., [36, 40]).

##### 3.1.3.3. Social/behavioural (*n* = 11)

Among the 11 studies that addressed social/behavioural difficulties, two were focused on juvenile arthritis (40, 44), one on muscular dystrophy (35), six on cerebral palsy (38, 48, 49, 50, 52, 53), and two on other conditions (28, 51). Among these 11 studies, 5 reported on rates of social/behavioral difficulties; specifically, juvenile arthritis (40), cerebral palsy (48, 53), and other conditions (28, 51), ranging from 16.0 to 27.3%. Specifically, symptoms in participants with juvenile arthritis, 16.0% (22); cerebral palsy, 18.8% to 27.3% (48, 53); and other conditions, 17.3% to 25.0% (28, 51). Several studies compared rates of social/behavioural difficulties in youth with physical disabilities to typically developing youth: some found that youth with cerebral palsy had greater levels of social/behavioural difficulties (e.g., [48, 52, 53]), or lower levels of behavior or conduct problems (38) whereas one study found no significant differences between youth with JIA and typically developing youth in some aspects such as behavioural psychological functioning (40).

##### 3.1.3.4. Mental health problems (not specified) (*n* = 9)

Among the 9 studies that addressed mental health problems (not specified), three were focused on spina bifida (27, 45, 47), one on juvenile arthritis (40), two on cerebral palsy (38, 54), and three on other conditions (28, 51, 55). Among these 9 studies, 6 reported on rates of mental health problems, specifically spina bifida (27, 47), juvenile arthritis (40), cerebral palsy (38), and other conditions (28, 51), ranging from 16.0% to 69.0%. Mental health problems in participants with spina bifida were 49.2% to 69.0% (27, 47); juvenile arthritis, 41.6% (40); cerebral palsy, 16.0% (38); and other, 32.2% to 50.8% (28, 51). When studies compared rates of mental health problems in youth with physical disabilities to typically developing youth using self-report, they did not find significant differences between groups (40, 45, 54, 55), however one study found that based on parental report, young people with CP have more mental health problems than youth in the general population (54).

##### 3.1.3.5. Attention deficit hyperactivity disorders (*n* = 6)

Among the six studies that addressed attention deficit hyperactivity disorders (ADHD), one was focused on muscular dystrophy (34), four on cerebral palsy (38, 48, 52, 53), and one on other conditions (51). Among these six studies, three reported on rates of ADHD, specifically muscular dystrophy (34), cerebral palsy (48), and other conditions (51), ranging from 16.7% to 19.7%. Reported symptoms in participants with muscular dystrophy were 16.7% (34); cerebral palsy, 19.5% (48); and other, 19.7% (51). When studies compared rates of ADHD in youth with physical disabilities to typically developing youth, one study found that youth with physical disabilities had lower levels of ADHD-related symptoms (38), one study found that youth with physical disabilities had increased hyperactivity and attentional difficulties (52), and another study did not find any difference in ADHD between the groups (48).

##### 3.1.3.6. Autism spectrum disorders (*n* = 3)

Among the three studies that addressed autism, one was focused on muscular dystrophy (29), one on cerebral palsy (56), and one on other conditions (51). Among these three studies, two reported on rates of autism symptoms, specifically, muscular dystrophy, 14.9% (29), and other, 3.3% (51), and one reported the rate of CP among people diagnosed with Asperger's syndrome (AS), 0.7% (56). Regarding the latter, Mouridsen et al. (56) did a retrospective study with a nationwide cohort of 4,180 people diagnosed with AS. Over an average observation period of 15 years, the authors discovered that the occurrence of cerebral palsy was 3 times higher for those with AS compared to those with cerebral palsy in the general population.

##### 3.1.3.7. Psychotic disorder (*n* = 2)

Among the two studies that addressed psychotic disorder, both were case studies focused on cerebral palsy (31, 50). For example, one of the studies described a 15-year-old female with cerebral palsy that was diagnosed with psychotic disorder (not otherwise specified) after experiencing four psychotic episodes within one year (31).

##### 3.1.3.8. Self-harm (*n* = 1)

Only one study reported on self-harm. It included a sub-sample of youth with spina bifida, and found a rate of 1.4% (57), however there was no higher or lower risk for self-harm associated with having spina bifida.

##### 3.1.3.9. Substance use and other health-risk behaviors (*n* = 1)

One cross-sectional study examining the prevalence of cigarette smoking, alcohol drinking, excessive drinking and illegal drug use among young adults with spina bifida found rates ranging from 11.0% to 92.0%, depending on the behavior. The authors concluded that substance use behaviors are less prevalent among those with spina bifida, but current alcohol consumption is more frequent in young adults with depressive symptoms (42).

#### 3.1.4. Experience of mental health problems and mental health services

Ten articles addressed the experience of mental health problems and/or mental health services access or use among young people with childhood-onset physical disabilities. Three out of the ten studies (40, 53, 55) used a mixed-methods approach that included qualitative interviews. Six out of the ten studies used qualitative methods only (31, 32, 35, 44, 49, 50), including semi-structured interviews and focus groups, and one study used sociodemographic information pertaining to mental health services received (36). In addition, most of these studies reported data on the entire sample (e.g., 2 papers reported on case studies of a young person with cerebral palsy and psychosis). Four themes were identified through our review of the findings from these studies: (1) access, use, and experiences of mental health services; (2) stigma; (3) mental health of family caregivers; and (4) importance of peer and vocational support services. We have organized the results from these studies in relation to these four themes in Table 5, and present a synthesis below.

**Table 5:** Experience of mental health problems and mental health services for young people with childhood-onset physical disabilities.

Theme	Studies^a^	Reported Results
Access, Use, and Experience of Mental Health Services	5. Brossard-Racine et al., 2013 8. Florou et al., 2016 9 Foster et al., 2010 10. Grody & Coffey, 2012 12. Hanson et al., 2018 15. Lindsay et al., 2017 17. Nicholls et al., 2015^b^ 22. Russo et al., 2012 32. Woodward et al., 2012	– Limited psychological services received within the past 6 months (15.4% overall and 24% for those with behavioural difficulties) – None of the children with spina bifida reporting clinically significant mental health concerns (anxiety and depression) were receiving mental health care – 18% of parents reported their child had an unmet mental health need in the past 12 months – Little formal help-seeking for psychological concerns at work, relied on co-workers or self – Delay in seeing psychiatrist until 1 year after onset of mental health concerns – Interventions perceived as helpful (peer support, vocational services) were not always available – Lack of access to vocational services in adult-oriented services (highlighted by young people, parents, and clinicians) – Lack of access to mental health services led to denial of symptoms and help-seeking delay – More hyperactive symptoms associated with increased likelihood of receiving mental health services – Clinicians, parents, and participants reported wanting more support for sexuality, relationships, and depression in adult oriented services – Accessed mental health services through a pediatrician – Accessed mental health services through pediatric emergency department clinic (previously sought help from a school psychologist) – 21% of participants were referred to a psychologist through the study – Treated with medication and therapy (CBT and supportive directive therapy) – Treated with medication
Stigma	8. Florou et al., 2016 12. Hanson et al., 2018 22. Russo et al., 2012	– Parental denial of handicap contributed to help-seeking delay – Decisions about disclosing arthritis at work caused anxiety, concerns about how employers would react – Adolescents reported not talking about their disease (JIA) with others out of shame or fear
Mental Health of Family Caregivers	8. Florou et al., 2016 27. Tong et al., 2013	– Mental health support for parents facilitated young person's progress – Mental health services for family members highlighted as an important component of care and service delivery
Importance of Peer and Vocational Support Services *Subtheme:* Peer Support	12. Hanson et al., 2018 15. Lindsay et al., 2017 27. Tong et al., 2013	– Peer support experienced as helpful – Clinicians identified peer support as potentially helpful – Peer support highlighted by nearly all participants to reduce isolation and share coping strategies and experiences
*Subtheme:* Vocational Support	12. Hanson et al., 2018 15. Lindsay et al., 2017 27. Tong et al., 2013	– Vocational support experienced as helpful – Vocational rehabilitation was highlighted as an important service – Suggestions to have combined clinics that include psychological and social services (vocational guidance, social aid)

^a^ For each study cited within this table, the numbers in the second column represent the selected studies presented in alphabetical order in **Supplementary File Table 2**: Overview of Studies Reviewed (1–33).

^b^ Data pertaining to access to services was collected through sociodemographic questions (e.g., received mental health services in the past, currently receiving mental health services, etc.).

With regards to the theme of access, use, and experiences of mental health services (31, 32, 35, 36, 40, 49, 50, 53, 55), studies highlighted that: few participants (i.e., less than a quarter the sample) that had mental health problems were receiving mental health care; a need for services to address issues such as sexuality, relationships, depression, peer support, and vocational services; a lack of formalized help seeking; delays in help seeking; and delays in receiving care. Studies describing how participants accessed mental health services (31, 40, 50) showed different pathways to care such as: pediatrician, pediatric emergency department clinic, and referral to psychologist. Details on the type of treatment and services that participants received was limited; however, two case studies (31, 50) mentioned medication for psychosis symptom treatment, cognitive behavioral therapy, and supportive directive therapy. Foster et al. (50) also highlighted that parents should be involved in the diagnosis of mental illness among children with cerebral palsy, should be educated about the mental illnesses their child is at risk for, and given tools on how to advocate for their child.

With regards to the theme of stigma (32, 40, 49), studies highlighted the influence of stigma experienced by parents, or the young people themselves, on delay in help-seeking, as well as its contribution to feelings of anxiety and decisions to disclose their disability to others. With regards to the theme of mental health of family and caregivers (44, 49), studies highlighted the importance of having mental health support and services for family members as a component of services for young people with disabilities. Regarding the theme of the importance for peer support and vocational support services (32, 35, 44): peer support was highlighted by clinicians and young people as a potentially helpful resource and a way to reduce isolation for young people with physical disabilities and vocational support was emphasized as being an important component of psychological and social services that young people with physical disabilities receive.

## 4. Discussion

This review sought to better understand the occurrence, experience of mental health problems, and mental health services access and use, among young people with childhood-onset physical disabilities.

### 4.1. Principal findings

Collectively, there was significant variation in the assessment measures and methods, source of reporting, and conceptualization of mental health problems, making it challenging to synthesize and compare results across studies. For example, we identified 35 separate measures that were used to assess mental health across 33 studies. Moreover, across the studies, authors used different sources of data, methods of data collection (e.g., self/parental report versus clinician report; interview or observation vs. self-report), and clinical cutoff points between measures. There was also variation in how authors approached the phenomenon of mental health problems, with some focusing on specific conditions (e.g., depression) and others used a non-categorical/broad approach (e.g., perceived mental health). Collectively, these factors may explain why we found a large variation in the reported rates of mental health concerns between the studies and even within a study. It may also explain why we found inconsistent results across the few studies that compared the occurrence of mental health problems in young people with childhood-onset physical disabilities to typically developing young people. In addition, differences in the prevalence of mental health concerns may be partly based on various sociodemographic and other clinical factors, such as the severity of physical conditions and related disabilities, which was not consistently detailed in the studies.

The three most common mental health concerns investigated in the literature were depression and mood-related difficulties, anxiety, and social and behavioural difficulties. The focus on depression and anxiety is concomitant with the prevalence of these disorders among young people in the general population (58). Social and behavioral difficulties were also commonly investigated, which may be attributed to the importance of social relationships among young people. Social elements such as belonging and having authentic friendships have been identified as contributing factors to meaningful participation in leisure activities among young people with disabilities (59). It is important to identify social difficulties early on to reduce their impact on participation and, consequently, the well-being of young people. Some of the factors associated with mental illness identified in the selected studies included impairments associated with the physical disability, pain, and family-related challenges. These factors are therefore important to consider when monitoring for symptoms of mental illness among young people with childhood-onset physical disabilities as they may help to identify individuals at risk for developing mental disorders.

We identified several themes from the qualitative data including: access, use, and experience of mental health services; stigma; mental health of family caregivers; and peer-to-peer and vocational support services. The theme of access, use, and experience of mental health services indicate that many young people experiencing mental health concerns (including clinically significant symptoms) are not accessing mental health services, and that young people and their families often experience several unmet psychological and social needs. Unmet mental health needs have been previously found to be higher in children with special healthcare needs that also have chronic emotional, behavioural, or developmental problems compared to children with special healthcare needs that do not have chronic problems of this nature (60). Needs that are unmet by mental health services in a timely manner can put young people at risk of deterioration, which may also impact their transition into adulthood. More research is needed to further understand access and use of mental health services and the reasons for limited access among young people with childhood-onset physical disabilities. For example, concerns around stigma related to the physical condition were highlighted in a few of the included studies (32, 40, 49) and may influence help-seeking. Another factor that may limit access is how services are organized for this population and the potential lack of coordination between physical rehabilitation settings and the mental health care system. Transportation challenges have also been highlighted as specific barriers in research (61). Technology (e.g., e-mental health services) may be able to play a role in removing some of the barriers that young people with physical disabilities face in accessing mental health services (62). The additional themes pertaining to the importance of peer and vocational support services and the mental health of family and caregivers indicate the need for a comprehensive psychosocial approach to providing mental healthcare to young people with childhood onset physical disabilities.

### 4.2. Identified gaps in current knowledge and future directions for research

Few studies explored the functional limitations stemming from comorbid disorders, therefore additional research on this topic is needed. Indeed, physical impairments have an impact on social roles and daily activities, which in turn affect mental health. While a few studies touched on quality of life, participation in activities were not well-addressed by the studies we reviewed. In addition, the selected articles were mostly cross-sectional studies; different types of study designs could improve the reliability of evidence for these populations. Furthermore, the studies in this review used a wide variety of measures to assess mental health, including non-validated tools. Future research using validated measures to assess mental health problems is warranted. This will not only improve the reliability and validity of results, but also will facilitate knowledge synthesis. Concurrently, guidelines for assessing the mental health status of young people with childhood-onset physical disabilities, including recommendations for multi-method and multi-informant (e.g., parents, youth, others) approaches to assessment, are important avenues for further consideration.

Across the studies, an array of factors was investigated or discussed in relation to mental health concerns among youth with physical disabilities, such as: physical, cognitive, and neurological impairments (27, 30, 31, 33, 36, 38, 49, 51, 52, 57), pain (4, 27, 38, 39, 41, 44, 48, 54), family-related factors, such as parental mental health problems, stress, anxiety, and support (27, 28, 39, 49, 50, 54), negative attitudes and negative attributes (37, 46), health-related quality of life (28, 43), sex (38, 39), social or vocational disability (32, 35), ability to ambulate (34), self-image (49), low birth weight (51), age (28, 53), isolation (44), steroid use (44), access to support for parents (49), social challenges at school (50), verbal status (i.e., being non-verbal) (52), and having spinal lesions of L2 and above (45). It was beyond the scope and objectives of this review to systematically review these factors; however, this is an area for future research attention. Such a review could provide youth, family members and service providers with insights on factors that can contribute to the mental health of young people with physical disabilities. This knowledge could help contribute to earlier identification of mental health problems among this population as well as an increased focus on preventative care.

Although we found a handful of studies examining mental health services access and use among our target population, this is a relatively nascent field that would benefit from more extensive health services research attention. Moreover, while some articles indicated the need for additional mental health (and comprehensive) services for young people with physical disabilities, there was limited attention in these studies on how to organize services for this population. A better understanding of the organizational contexts of various healthcare systems or models of service delivery would be pertinent to ensure the complex needs of this population are met. It is important to consider the needs of this population within the global efforts already underway to transform youth mental health service delivery.

### 4.3. Strengths and limitations

This review has both limitations and strengths. In terms of limitations, for feasibility purposes, we restricted the search from 2007 onwards, which limits the body of evidence reviewed. A study quality assessment was not done due to the large variability of study designs and research approaches (further detailed below); this is also not a requirement of conducting scoping reviews as the objective is to map the evidence on an emerging topic. Moreover, the summaries of reported mental health prevalence rates should be interpreted with caution, given the variability of study designs, sampling methods, populations, measures, missing information, and the lack of quality assessment. Additionally, calculated means were not weighted based on the study sample sizes; as such, these values are likely skewed due to rates from smaller and larger sample sizes equally contributing to the mean. We also calculated unweighted medians (i.e., median values could be skewed by outliers). We decided to report on the overall mean and median prevalence rates for the purposes of summarizing the data in the context of a scoping review, however, these should not be taken as conclusive estimates. Further, to reduce misclassification of the summarized prevalence rates, we did not consider borderline cases in our summaries (as these were not reported consistently within the selected studies). In addition, the articles about health risk behaviours or risk factors for psychiatric disorders were included, but their contribution was limited to answer the study question.

However they were included as being of interest to present issues that are related to mental and physical health in this population. These articles help to highlight behaviours that should be considered by healthcare professionals when interacting with young people with disabilities. In the majority of the studies, the entire sample included youth with physical disabilities (e.g., cerebral palsy, spina bifida, muscular dystrophy, etc.); however, some studies had a mixed sample or took on a non-categorical approach, which are important to consider when interpreting these results. In addition, the data on lived experiences of mental health problems and mental health services access and use was obtained from a heterogenous range of studies, for example, in terms of clinical population, percentage of sample known to be experiencing mental health problems, and data collection method.

In terms of strengths, we used a comprehensive search strategy to increase the likelihood of identifying relevant studies. For example, given that many mental illnesses develop during young adulthood, and many young people may not have access to formal diagnostic procedures, we used broad eligibility criteria to describe psychological issues, mental illnesses, or symptoms of mental illness. Moreover, the key words for the search strategy were built on a previously published scoping review (18) which helped with the comprehensiveness of the search terms. In addition, minimal exclusion criteria related to study design additionally allowed us to synthesize both quantitative and qualitative data which contributed to our objective of obtaining a portrait of the extent and nature of the literature. To increase the rigor of this review, we collaborated with the rehabilitation school librarian who helped apply a systematic search strategy and based the protocol on well-established guidelines for conducting scoping reviews (14, 15, 16). A data extraction process involving an additional reviewer auditing the data extraction of all articles, and validation of the extracted data by other members of the research team contributed to the rigor of the review. Qualitative synthesis of themes was also validated by an additional reviewer and reviewed with a third member of the team.

## 5. Conclusions

This scoping review shows that mental health problems are common in adolescents and young adults with childhood-onset physical disabilities. It also highlights the need for adapting healthcare services to increase accessibility and use of mental health supports, and identifies methodological and research design gaps in the literature. Findings suggest the importance of healthcare professionals being aware of the mental health needs of young people with childhood-onset physical disabilities, and the need for services research on how best to address their access to mental health services.

## Data Availability

The original contributions presented in the study are included in the article/**Supplementary Material**, further inquiries can be directed to the corresponding author/s.
