# Supplementary material for: Mental health problems among adolescents and young adults with childhood-onset physical disabilities: A scoping review
Source: Front Rehabil Sci. 2022 Sep 6;3:904586. doi: 10.3389/fresc.2022.904586 (PMC9485587; doi:10.3389/fresc.2022.904586)
Supplement: Supplementary file 2 [file Table_2_v1.docx]

**Supplementary File 2.** Overview of studies reviewed

|  | Study characteristics  (author, year, title, country) | Study objective | Study population^^[[1]](#footnote-1)^^ and  research design | Methods and measures^^[[2]](#footnote-2)^^ | Results |
| --- | --- | --- | --- | --- | --- |
| 1 | Abdul-Sattar et al., 2014  Determinants of health-related quality of life impairment in Egyptian children and adolescents with juvenile idiopathic arthritis  Egypt | To identify factors related to health-related quality of life (HRQoL) for children and adolescents with juvenile idiopathic arthritis (JIA) | 58 youth with JIA (17 males, mean age = 9.1, range = 7-17) recruited from in- and outpatient rheumatology clinics  Cross-sectional study | Children’s Depression Inventory (CDI)  Pediatric Quality of Life Inventory (PedsQL) | - 36% (n=21/58) of youth with JIA had levels of depression symptoms considered to be clinically significant as measured by the CDI (T-score ≥ 70)  - Higher scores of depressive symptoms were associated with lower HRQoL in both univariate (OR: 2.5, 95% CI 1.7-5.9, P = 0.02) and multiple regression (OR: 4.2, 95% CI: 2.0-12.6, P = 0.001) analyses |
| 2 | Adegboye et al., 2017  Theory of mind, emotional and social functioning, and motor severity in children and adolescents with dystonic cerebral palsy  United Kingdom (UK) | To investigate the degree of social and emotional difficulties in children and adolescents with dystonic cerebral palsy (CP) and communication difficulties | n= 22 youth with CP (12 males,  mean age= 13, range= 8-17, 88% dystonic CP)  recruited from a tertiary hospital and a specialized school for children with disabilities in the UK  n= 20 typically developing (TD) youth (10 males, mean age= 12, range= 8-16) recruited from youth groups  Cross-sectional study | Emotion Regulation Checklist (ERC)  Strengths and Difficulties Questionnaire (SDQ)  [both parent report]  The Reading the Mind in the Eyes Test (RMET) - to assess Theory of Mind (ToM)  Ravens Standard Progressive Matrices (RSPM) OR Perceptual Reasoning Index subtests of the Weschler Scales of Intelligence IV (WSI IV) - to assess IQ | - CP group had significantly lower RMET scores and more social difficulties than TD group  - No significant differences in emotional regulation ability in CP versus TD group  - Nonverbal participants had lower RMET scores than verbal participants, and exhibited greater social impairment  - CP individuals (particularly if nonverbal) showed increased difficulties on the ‘hyperactivity and attentional difficulties’ subscale of the SDQ (marginally significant)  - Emotional regulation ability and hyperactivity and attentional difficulties were found to mediate the relationship between ToM and social functioning  - Lower IQ and motor ability was associated with lower RMET scores  - Males with CP exhibited significantly higher levels of social difficulty than females with CP |
| 3 | Bellin et al., 2010  Correlates of depressive and anxiety symptoms in young adults with spina bifida  USA | To examine the relationship between ecological factors and psychological symptoms for youth with spina bifida (SB) | n= 61 young adults with SB (24 males, mean age = 21, range= 18-25, 82% with myelomeningocele) recruited from 5 SB clinics  Cross-sectional study | Child Attitude Towards Illness Scale (CATIS)  Hopkins Symptom Checklist (HSCL-25)  SB severity composite - to measure pain and severity of SB  Family APGAR - to measure satisfaction with family interaction  Patient Assessment of Chronic Illness Care (PACIC) | - 49% of young adults with SB reported psychological symptoms above clinical cut-off  - 41% fell into clinical range for depressive symptoms  - 31% reported scores above clinical cut-off for anxiety symptoms  - More positive attitude towards SB and family functioning were associated with lower levels of depressive symptoms  - Higher level of pain was associated with higher levels of anxiety symptoms |
| 4 | Briegel et al., 2010  Behaviour problems of patients with Moebius sequence and parental stress  Germany | To investigate behavior problems of youth with Moebius sequence, their parents’ life satisfaction and any potential correlations between the two factors | n = 31 youth with Moebius sequence (15 males, median = 10 years, 7 months, range= 4-17; 4-11 years (n=19);  12 to 17 (n=12) recruited from the German Mobius foundation  Cross-sectional study | Child Behaviour Checklist (CBCL)  Social Orientation of Parents with Handicapped Children questionnaire (SOEBEK) - to measure parental stress, given to parents of patients 1-14 years old | - 1 out of 12 girls between ages 12 to 17, was in the clinical range on the anxious/depressed CBCL score  - Parental stress had a significantly moderate correlation with anxious/depressed behaviour, aggressive behaviour, externalizing problems, and total problem score on the CBCL  -Social problems were more prevalent among the 12–17-year-old group, 3/12 (25%) were clinically significant  - 32.2% of subjects (7 males, 3 females) were in the clinical range on at least one of the CBCL scales  - 9.7% (2 males, 1 female) had a total problem score within the clinical range |
| 5 | Brossard-Racine et al., 2013  Behavioural difficulties in adolescents with cerebral palsy  Canada | To describe the behavioural patterns present in CP and explore associated factors | n = 160 adolescents with CP (95 males, mean age = 15.4 ±2.17)  recruited from clinics, rehabilitation centers, schools, community,  and transition programs across the province of Quebec  Cross-sectional study | Strengths and Difficulties Questionnaire (SDQ-25)  Vineland Adaptive Behavior Scales-Interview- Second Edition    [both parent report] | - 15.4% of participants had received psychology services in the past 6 months  - Behavioural difficulties remained common among adolescents with CP (37%), 18.8% in the clinically abnormal range, and 18.1% in the borderline range  - Peer problems were most frequent for youth with CP, followed by emotional symptoms, conduct problems, hyperactivity, and lack of prosocial behaviors  - Adolescents with CP were more likely than TD peers to have behavioural difficulties (with the exception of conduct problems)  - Younger participants had worse emotional conduct, hyperactivity and more difficulty than older participants  - More hyperactive symptoms were associated with an increased likelihood of having received psychological services in the past 6 months  - Of those with behavioural difficulties, only 24% received psychological services within the past 6 months |
| 6 | Colombo et al., 2017  Assessing mental health in boys with Duchenne muscular dystrophy: Emotional, behavioural and neurodevelopmental profile in an Italian clinical sample  Italy | To describe the psychopathological, emotional, behavioral, and neurodevelopmental profile of boys with [Duchenne Muscular Dystrophy](https://www-sciencedirect-com.proxy3.library.mcgill.ca/topics/medicine-and-dentistry/duchenne-muscular-dystrophy) (DMD)  To investigate the relationship between their psychopathological profile and neurological phenotype | 47 males with DMD (mean age = 10.96, range= 2–18) recruited from the neuromuscular unit of a university health center  Cross-sectional study | Child Behaviour Checklist (CBCL)  Youth Self Report (YSR)  Strengths and Difficulties Questionnaire (SDQ) [parent and youth forms]  Autism Diagnostic Observation Schedule (ADOS)  Weschler Intelligence Scale (WISC-III or WIPPSO) or Griffiths scale (based on age) | - Regarding CBCL t scores, border and clinical cases were most commonly found for internalizing problems scale (4 border, 11 clinical); withdrawn/depressed subscale (7 border, 3 clinical); anxious/depressed subscale (7 border, 1 clinical)  - 8.5% (n=4/47) clinical cases for depression; and 2% (n=1/47) for anxiety  - CBCL withdrawn/depressed subscale scores were significantly higher for those using a wheelchair and those with intellectual disability  - 7/47 of the boys with DMD had a diagnosis of ASD |
| 7 | Essner & Holmbeck, 2010  The impact of family, peer, and school contexts on depressive symptoms in adolescents with spina bifida  USA | To determine the impact of SB on the relationship between positive experiences across contexts (family, school, peers) and depression symptoms | Study included data from fourth and fifth waves of data collection  Time 4: n = 125 (n=60 families of adolescents with SB, n = 65 matched comparisons) (mean age = 14.57, range = 14-15)  Time 5: n=111 (n= 52 families of adolescents with SB, n=61 matched comparisons (mean age = 16.64, range = 16-17) from 2 children’s hospitals, a university-based medical centre, and a SB association  Longitudinal two-wave study | Child Behaviour Checklist (CBCL) - social competence subscale - parent report  Harter’s Rating Scale of Child’s Actual Behaviour - parent/teacher report  Child Depression Inventory (CDI) | - In comparison to the TD group, youth with SB had lower total levels of positive experiences across all contexts (combined family, school, and peer experiences)  - Having positive experiences across contexts was not a significant determinant of depressive symptoms for youth with SB aged 14–15 compared to the TD group; specifically, positive peer context was associated with decreased depression symptoms in the TD group whereas depression symptoms did not change for the SB group  - Longitudinal effects were not found for any of the associations between positive contexts and depressive symptoms  - In adolescents with SB, higher levels of positive school experiences were found to be protective against the effects of lower levels of positive peer experiences on depressive symptoms |
| 8 | Florou et al., 2016  Working through physical disability in psychoanalytic psychotherapy with an adolescent boy  Greece | To describe a psychoanalytic process of therapy conducted with an adolescent male with CP | n = 1, 15-year-old male with CP  Case study of youth with CP having first time access to mental health services, specifically weekly psychoanalytic therapy for 1 year | Psychiatric interview and psychoanalytic psychotherapy including illustrations of transference and countertransference | - Denial from the parents about having a handicapped child due to lack of access to mental health services, did not seek help until age 15 (because child felt rejected by peers)  - Lack of psychological development (disorganized speech and thoughts) due to lack of support, and denial of disability  - Exhibited experiences of sadness, anxiety, feelings of persecution, sadistic, and masochistic tendencies  - Identified as weak person and considered himself defective and not worthy of living  - Parents received additional counselling to work through their problems (mother’s overprotectiveness, and father’s aggression) which facilitated progress |
| 9 | Foster et al., 2010  Psychiatric complications in cerebral palsy  USA | To provide information regarding psychiatric complications associated with  CP | n = 1, 15-year-old female in 10th grade with spastic diplegia CP, with many episodes of psychosis  Case study | Mental Status Examination and psychiatric interview | - Referred to clinic due to four psychotic episodes within the past year  - Symptoms included: changes in sleep pattern, seeing things, inappropriate laughter, aches, irritability, extreme mood shifts, insomnia, paranoid delusions/statements, bizarre speech  - First sought help from her pediatrician, then hospitalized for acute change in mental status  - Did not see psychiatrist until nearly a year after first episode where she was diagnosed with psychotic disorder not otherwise specified (NOS)  - At the study clinic she was diagnosed with a mood disorder (due to probable seizure disorder), abnormal EEG, social isolation  - Recommended to continue antipsychotic and anticonvulsant medication and to receive interventions including components of supportive directive therapy and cognitive-behavioral therapy |
| 10 | Grody & Coffey, 2012  Presentation and treatment of acute psychosis in an adolescent girl with cerebral palsy  USA | To describe diagnostic and treatment challenges when faced with acute-onset psychotic symptoms in an adolescent with CP | n=1, 17-year-old girl with spastic quadriplegia CP and global developmental delay recruited from a pediatric emergency department  Case study | Mental Status Examination and psychiatric interview | - Presented to the clinic with acute psychotic symptoms (disorganized speech and behavior, paranoid delusions, auditory hallucinations)  - Psychosocial stressors prior to onset of illness (challenges to self-esteem at school, academically and socially)  -Possible anxiety regarding her family’s continuing ability to care for her  - No psychiatric history, previously evaluated by school psychologist around self-esteem issues  - Diagnosed with psychotic disorder NOS, treated with antipsychotics |
| 11 | Hanns et al., 2018  Depressive symptoms, pain and disability for adolescent patients with juvenile idiopathic arthritis: results from the Childhood Arthritis Prospective Study  UK | To investigate whether depressive symptoms measured around the time of JIA diagnosis are associated with future pain, disability and diseases among adolescents | n= 102 JIA patients (44 males, median age= 13.2, range= 11.9–14.2) recruited to the Childhood Arthritis Prospective Study  Longitudinal study | Mood and Feelings Questionnaire (MFQ)  Childhood Health Assessment Questionnaire (CHAQ)  Pain and general evaluation of disease - visual analogue scale (VAS) | - 14.7% had symptoms over the clinical cut-off for major depressive disorder at baseline  - Adolescents with JIA and depressive symptoms had more active and limited joint counts, pain and disability at baseline  - Associations between baseline depression and both pain and disability continued for at least one year  - From 12 to 48 months, estimated disability and pain scores were significantly worse for those with high scores for depressive symptoms at baseline |
| 12 | Hanson et al., 2018  Experiences of employment among young people with juvenile idiopathic arthritis: a qualitative study  UK | To investigate the employment experiences and expectations of youth living with JIA and to better understand health professionals’ role in employment outcomes | n = 29 young adults with JIA (10 males, median age = 22, range= 16-31) and  n = 9 healthcare professionals  recruited from three UK teaching hospitals with adult JIA services  Qualitative descriptive study | Participants - JIA:  - Semi-structured interviews  - Focus groups  Participants - Health Professionals:  - Semi-structured interviews | - Participants with JIA had limited knowledge regarding their rights under antidiscrimination laws and concerns about employers’ attitudes towards, and willingness to support employees with long-term health conditions (e.g., offering accommodations such as frequent breaks)  - Participants with JIA reported experiencing psychological impacts from JIA such as anxiety, mood effects and altered body image; they rarely sought formal support and often relied on support from co-workers or “suffered in silence”  - Decisions about disclosing arthritis caused anxiety  - Psychosocial and vocational interventions (including peer support) were experienced as helpful (e.g., for social and emotional wellbeing) but not always available |
| 13 | Kelly et al., 2012  Executive functioning and psychological adjustment in children and youth with spina bifida  USA | To assess executive functioning and psychological outcomes in a group of children and adolescents with SB compared to a TD control group | n = 51 adolescents with spina bifida (22 males, mean age = 13, range= 10-17)  Controls, n = 45 (21 males, mean age = 11.8, range= 10-16) recruited from 2 Ohio SB clinics (SB youth), and pediatrician’s offices and advertisements (controls).  Cross-sectional study | Behavior Rating Inventory of Executive Function (BRIEF) - mainly the Metacognition Index scale [parent rating]  Behavior Assessment System for Children (BASC-2) [parent rating scale]  Child Depression Inventory (CDI) [both youth, CDI- Child, and parent report, CDI- Mother, versions]  Children’s Loneliness Scale | - SB group had greater difficulties than controls at internalizing symptoms, adaptive functioning and depressive symptoms as reported by mothers (CDI- Mother)  - Youth with SB did not report more externalizing problems, depressive symptoms (CDI - Child) or loneliness than controls  - The Metacognition Index Scale (measure of executive functioning) completely mediated the relationship between group status, internalizing problems and depressive symptoms (CDI-Mother), but only partially mediated the relationship between group status and adaptive functioning |
| 14 | Latimer et al., 2017  Secondary conditions among males with Duchenne or Becker muscular dystrophy  USA | To report a broader range of secondary conditions by phenotype of muscular dystrophy (MD) and evaluate whether frequencies differ by ambulation status | n = 209 caregivers of males with MD (mean age= 17, range = 3-31) recruited from 6 sites affiliated with a MD research network  Cross-sectional study | Family Quality of Life Survey | - 28% (53/192) reported depression symptoms  - 5 most commonly reported conditions in males with DMD were cognitive deficits (38%), constipation (32%), anxiety (29%), depression (27%), and obesity (20%)  - Among males with the Becker phenotype, the most commonly reported conditions were cognitive deficits (36%), depression (29%), attention deficit hyperactivity disorder (ADHD, 32%), constipation (21%), and trouble holding urine (21%)  - Higher frequencies of anxiety, depression, and kidney stones were found among non-ambulatory males  - ADHD was more common in ambulatory males (32%) |
| 15 | Lindsay et al., 2017 Enablers and barriers of men with Duchenne muscular dystrophy transitioning from an adult clinic within a pediatric hospital Canada | To investigate experiences of clinicians, young men, and parents during the transition from an adult DMD clinic within a pediatric hospital to an adult health facility and better understand the enablers and barriers during the transition process | n = 16 participants (7 clinicians, 5 parents, 4 young adult males with DMD, mean age=23.5, range= 20-28) recruited from an adult DMD clinic at a pediatric hospital  Qualitative study | Semi-structured, in-depth interviews | - Isolation and depression were prominent after completing school (theme brought up as a challenge by clinicians, parents and youth)  - Findings highlighted the importance of occupational rehabilitation (post-secondary education, volunteering, and work)  - Youth, parents, and clinicians discussed lack of support for social, educational, and vocational needs in the adult system as a barrier  - There is a want for more support for sexuality, relationships and depression in adult-oriented OT  - Youth could benefit from peer mentoring sessions or counselling support to help with depression and loneliness (mentioned by clinicians) |
| 16 | Mouridsen et al., 2013  Cerebral palsy in individuals with a history of Asperger’s syndrome— A Danish nationwide register study based on hospital diagnoses  Denmark | To compare rates of CP in a large nationwide cohort of people diagnosed with Asperger’s Syndrome (AS) with the general population | n= 27 youth with CP (21 males, mean age= 18.1, range =4-31) from a total of 4180 with AS (3431 males) recruited from the Danish National Health Register (DNHR)  Retrospective longitudinal study | Screening of DNHR data | - 27 patients with AS were found with a diagnosis of CP  - The prevalence is more than 3x higher than in the general population  - Unspecified subtype of CP was particularly common among individuals diagnosed with AS (CP in 61.9% of males, and 66.7% of females with AS) |
| 17 | Nicholls et al., 2015  Psychological functioning in youth with spina bifida living in Colombia, South America  Columbia | To determine if SB impacts self-reported symptoms of depression and anxiety | n= 22 youth with SB (15 males, mean age = 13, range= 8-17) recruited from a university teaching hospital,  n=22 controls with typical development (15 males, mean age= 13) recruited through flyers posted at churches, stores, and restaurants.  Cross-sectional study | Children’s Depression inventory (CDI)  Revised Children’s Manifest Anxiety Scale (RCMAS-2) | - There were more depressive symptoms among the SB group (t(42)=-2.38, p=.02, d=0.71, 95% CI=-1.13 to -0.09)  - About 14% of children with SB fell within the clinically significant range for depressive symptoms (versus 0 control children)  - Children with SB had significantly lower anxiety scores versus control children (t(43) = 2.80, p = .04, d = 0.63, 95% CI = 0.06-4.12)  - 22.72% of children with SB fell within the clinically concerning range for anxiety scores, versus 31.82% of control children however the likelihood of being in the clinically concerning range did not differ between groups  - Social anxiety scores did not differ between SB and control groups  - 18% of children with SB fell in the clinically concerning range for social anxiety, compared to 4.54% of controls, however the likelihood of being in the clinically concerning range did not differ between groups  - None of the children with SB who reported clinically significant scores for anxiety and/or depression were receiving mental health care |
| 18 | Ramsey et al., 2013  A prospective examination of attitudes toward illness and depressive symptoms in youth with Juvenile Rheumatic Diseases  USA | To investigate the association between attitudes towards illness and symptoms of depression among young people with juvenile rheumatic diseases (JRDs) prospectively | n = 30 youth with JRD (8 males, mean age = 12.9, range= 7–18) recruited from a pediatric rheumatology clinic  Longitudinal study | Child Attitude Toward Illness Scale (CATIS)  Children’s Depression Inventory (CDI) | - Children’s negative attitudes toward illness at baseline were significantly associated with depressive symptoms at 12 months follow-up (r=-0.76, p < 0.01) |
| 19 | Ramstad et al., 2012  Mental health, health-related quality of life and recurrent musculoskeletal pain in children with cerebral palsy 8–18 years old  Norway | To investigate mental health and mental HRQoL among children with CP experiencing recurrent musculoskeletal pain using children’s self-report | n= 83 youth with musculoskeletal pain and CP (41 males, mean age = 14.2, range= 8-20) recruited from a university hospital  Cross-sectional study | Strengths and Difficulties Questionnaire (SDQ-25) [both child and parent form]  Pediatric Quality of Life (PedsQL) 4.0  Child Health Questionnaire (CHQ) pain questions  (Norwegian version)  General Health Questionnaire (GHQ- 30) (used to assess parents mental health) | - Parent reports, but not youth self-reports indicated that children with CP have more mental problems  - Both parent and children report indicated lower HRQoL in CP children compared to TD youth  - Greater scores on the SDQ peer problem scale were reported among children with recurrent musculoskeletal pain (self-report)  - Self reports indicate that recurrent musculoskeletal pain and female gender were associated with more mental problems  - Recurrent musculoskeletal pain and increased motor impairment was associated with lower HRQoL scores  - Parent reports indicated that more mental problems in parents were associated with more mental problems among children and lower HRQoL scores |
| 20 | Ramstad et al., 2015  Self-reported mental health in youth with cerebral palsy and associations to recurrent musculoskeletal pain  Norway | 1) To investigate self-reported emotional, conduct, hyperactivity, and peer problems among youth with CP in comparison to those without  2) To compare mother’s reports on mental health problems among children with CP to youth without CP of a similar age  3) To investigate the potential associations between self-reported mental health issues among children with CP and recurrent musculoskeletal pain | n=81 youth with CP (40 males, mean age = 14.2, range = 8-18) recruited from a defined geographic area and a tertiary hospital  Cross-sectional study | Structured interview on pain  Strengths and Difficulties Questionnaire (SDQ-25 Norwegian version) [Parent and child form] | - 67% reported musculoskeletal pain, and those reported higher levels of peer problems (in girls only) and higher total difficulty score compared to children without CP  - Compared to TD youth, children with CP reported higher levels of emotional problems (statistically significant for boys only) and lower levels of hyperactivity and conduct problems  - Ten participants had a score indicating a borderline risk of having psychiatric disorder  - 3 scored high risk of psychiatric disorder to Norwegian TDY  - All 13 participants at risk of having a psychiatric disorder had musculoskeletal pain  - Parents and youth reported hyperactivity differently |
| 21 | Rapp et al., 2017 Predictors of parent-reported quality of life of adolescents with cerebral palsy: A longitudinal study Germany, France, Italy, Denmark, Sweden, UK | To investigate whether QoL in adolescence can be predicted by childhood factors, family, personal factors of the parent, and/or changes between childhood and adolescence in pain, psychological issues, and parenting stress | n = 551 youth with CP (316 males, mean age = 15.1, range 12-18.6) from the SPARCLE European cohort of children with CP  Longitudinal study | KIDSCREEN-52  Child Health Questionnaire (CHQ) pain questions  Strengths and Difficulties Questionnaire (SDQ)  Parenting Stress Index Short Form (PSI)  [parent report] | - Impairment severity, family characteristics and socio-demographic factors were not predictors of lower adolescent QoL  - Female gender had a small negative effect on moods and emotions and self-perception  - Pain, psychological problems and parenting stress were predicted lower adolescent QoL in most domains |
| 22 | Russo et al., 2012  Psychological profile in children and adolescents with severe course Juvenile Idiopathic Arthritis  Italy | To investigate the psychosocial functioning of children and adolescents with JIA and the disease-related changes in their family | n = 33 youth with JIA (10 males, mean age = 10.5, range= 6–16) recruited from a tertiary care rehabilitation unit  (Group 1: Children group, n=16, range: 6-10 years)  Group 2: Adolescent group, n = 17, range = 11-16 years)  Cross-sectional study | Anxiety Checklist for Children and Adolescents  Children’s Depression Inventory (CDI; Italian version)  Achenback’s Child Behaviour Checklist 6-18 years (CBCL)  Open-ended interviews with parents and patients | - Self-reported psychological functioning (depression, anxiety, and behavior) was not different from the normal population with no psychopathology found; however, using clinical interviews significant psychological suffering was detected in all of the children group and 41.6% of the adolescent group (e.g., emotional lability was most commonly demonstrated in 33.3% of the adolescent group  - 21% (7/33) were referred to a psychologist  - JIA was a disrupting event causing changes in family QoL  - 41.6% in the adolescent group report the disease affects body image  - Social competence was below normal range in both children and adolescents  - 25% of adolescents reported not talking about their disease with others out of shame or fear of discrimination |
| 23 | Sienko, 2018  An exploratory study investigating the multidimensional factors impacting the health and well-being of young adults with cerebral palsy  USA | 1) To investigate whether functional motor ability among young adults with CP affects physical impairments (ambulatory decline, pain, pain interference, depression, fatigue), psychological (health locus of control), and social (emotional support) factors, overall health status and satisfaction with life.  2) To investigate the impact on overall health status and satisfaction with life from physical impairments, psychological and social factors. among young adults with CP. | n = 97 young adults with CP (47 males, mean age = 23.8, range = 18-30,  and n= 40 proxy report)  Recruited from hospital and rehabilitation center databases  Cross-sectional study - survey | Gross Motor Function Classification System (Expanded & Revised) - GMFCS (E&R)  Behavioural Risk Factor Surveillance System (BRFSS)    Impact of Pain and Functioning and Well-Being Scale of the Brief Pain Inventory (BPI)  Fatigue Assessment Scale (FAS)  Patient Health Questionnaire-9 (PHQ-9) (used to measure severity of depression) | - 42% of young adults with CP reported experiencing depression (25%, mild; 13%, moderate; 4%, severe)  - 77% reported they received the emotional support they needed usually or always, no difference found by GMFCS level  - Pain, fatigue and depression were reported in a large proportion of the sample for all levels of GMFCS  - For young adults with CP, emotional support plays a significant role in satisfaction with life |
| 24 | Singhal et al., 2014  Risk of self-harm and suicide in people with specific psychiatric and physical disorders—comparisons between disorders using English national record linkage  UK | To investigate associations between specific psychiatric and physical illnesses and self-harm | n = 14,563 with SB (6262 males) in a large national sample divided into 4 age groups  n = 34 youth with SB (range= 10-24) that had an episode of self-harm, recruited from linked English Hospital Episode Statistics and mortality datasets  Retrospective cohort study design | Episodes of self-harm for people hospitalized from 1999 to 2011 in English hospitals | - Cystic fibrosis, celiac disease, Crohn’s disease and SB were associated with a neither high nor low ratio of rate (RR)  - SB showed a reduced RR (0.70) in the 10–24-year-old group and an increased RR (1.90) in the 45–64-year-old group  - The prevalence of self-harm is higher among people with psychiatric (depression, anxiety, schizophrenia, substance abuse, eating disorders, schizophrenia) or physical disorders (epilepsy, asthma, migraine, psoriasis, diabetes mellitus, eczema, and inflammatory polyarthropathies) |
| 25 | Soe et al., 2012  Health risk behaviors among young adults with spina bifida  USA | To explore the prevalence of a health risk behavior (e.g., substance use) by age, and examine their association to depression for young adults with SB | n = 130 young adults with SB (64 males, mean age= 23, divided into three groups: 16–19, 20–24, 25–31, 64% lumbosacral lesion, 77% with shunt) recruited from the Arkansas Spinal Cord Commission (ASCC)  Cross-sectional study | Population-based survey conducted by ASCC in 2005, followed by an interview and self-  administered questionnaire on substance use and sexual behaviors  Centre for Epidemiological Studies Depression Scale (CES-D) | - Youth with SB reported depressive symptoms at a rate higher than the national estimate of depression among individuals within the same age group in the general population (48% vs 10%)  - Major depressive symptoms were associated with current alcohol drinking (odds ratio (OR): 4.74; 95% CI= 1.18-19.04)  - Respondents were less likely to use substances than TD youth (28% vs 35% for tobacco use, 28% vs 57% for alcohol use, 3% vs 17% for marijuana use)  - Nearly 90% visited a doctor in the past year  - Youth with SB tended to eat less healthy (75% of SB youth vs 12% TD youth report not eating healthy), do less exercise (74% SB youth vs 22% TD youth report not exercising), and engage in sedentary activities (83% SB youth vs. 39% TD youth report watching TV >3 hours/day) |
| 26 | Stevanovic & Susic, 2013  [Health-related quality of life and emotional problems in juvenile idiopathic arthritis](https://mcgill.on.worldcat.org/detailed-record/7021559038?databaseList=283&databaseList=638&scope=wz:12129)  Serbia | To evaluate the associations between levels of depression and anxiety symptoms and HRQoL in children with JIA and to examine these associations while considering demographic information, clinical factors, and pain | n = 67 children with JIA (19 males, mean age= 13.6, range= 8-18) and one parent per child, recruited from a rheumatology institute  Cross-sectional study  Note: Young people with severe mental health problems (e.g. psychotic disorders) were excluded from the sample | Serbian Screen for Child Anxiety Related Emotional Disorders Questionnaire (SCARED)  Mood and Feeling Questionnaire (MFQ)  Pediatric Quality of Life Inventory (PedsQL) | - Depressive symptoms, but not anxiety, accounted for substantial variability in levels of HRQoL when considered with demographics (gender, age, school achievement), clinical factors (JIA activity, age at JIA onset, JIA duration), and pain |
| 27 | Tong et al., 2013  [Consumer Perspectives on Pediatric Rheumatology Care and Service Delivery: A Qualitative Study](https://mcgill.on.worldcat.org/detailed-record/7007835171?databaseList=283&databaseList=638&scope=wz:12129)  Australia | To elicit parental and adolescent perspectives on pediatric rheumatology care and service delivery and to describe the impact of this process on a proposed model of care addressing pediatric rheumatology service delivery | n = 13 adolescents with JIA (4 males, mean age= not reported, range= 14-20), and 37 parents,  recruited from an arthritis network, 2 children’s hospitals, and pediatric rheumatologists’ private practice  (50 total participants; 37/50 participants were parents)  Qualitative study | Focus groups and semi-structured interviews, including 9 telephone and 4 face-to-face interviews with adolescents | - Five main themes including psychosocial (social/financial aid, mental health services, vocational guidance, peer) and school-based support and advocacy (to promote acceptance, academic assistance, environmental modifications)  - Mental health services identified specifically to address chronic pain and depression, mood and aggression related to steroid use, and low self-esteem in patients (as well as sibling support and family functioning)  - Almost all participants highlighted peer support as a way to share experiences/coping strategies and reduce isolation  - To improve quality of care, participants suggested combined clinics that included psychological and social services (vital to address chronic pain and depression, mood and aggression related to steroid use, low self-esteem) |
| 28 | Verhoef et al., 2007  Perceived health in young adults with spina bifida  The Netherlands | To investigate the perceived health of youth with SB in comparison with TD youth and understand the association between disease characteristics and health impairments | n= 164 with SB (72 males, mean age = 20 years 8 months, range = 16–25, and 115 with hydrocephalus) recruited from 11 SB clinics, rehabilitation centers, special housing facilities, special schools, the Dutch Association of Patients with SB, and magazine advertisements  Cross-sectional study | Medical Outcome Study short-form Health Survey (SF-36) Dutch version | - Young adults with SB perceived that they have poorer physical health than the general population  - Their perception of mental health did not vary from the reference group  - Participants with SB occulta had lower perceived heath than the general population on all SF-36 domains (including Role emotional and Mental health domains) and lower perceived health than participants with SB aperta with or without hydrocephalus on all SF-36 domains except for physical functioning  - Individuals with L2 and above lesions had significantly lower perceived mental health than individuals with L3-L5 and S1 and below lesions |
| 29 | Wagner et al., 2007  A Cognitive Diathesis-Stress Model of depressive symptoms in children and adolescents with Juvenile Rheumatic Disease  USA | To test a cognitive diathesis-stress theory of depressive symptoms (learned hopelessness theory) in children with JRD | n = 50 youth with JIA (n = 29), systemic lupus erythematosus (n = 12), juvenile dermatomyositis (n = 7), or juvenile ankylosing spondylitis (n = 2)  (19 males, mean age = 13.7, range= 9-17) recruited from a pediatric rheumatology clinic in a teaching hospital  Cross-sectional study | Children’s Depression Inventory (CDI)  Perceived control over daily illness symptoms (1 question Likert scale)  Children’s Attributional Style Questionnaire- Revised (CASQ-R) | - General negative attributions were associated with greater depressive symptoms only under conditions of low perceived control, after controlling for disease and demographic variables |
| 30 | Wagner et al, 2015  Perception of secondary conditions in adults with spina bifida and impact on daily life  USA | To describe the perception of secondary conditions in individuals with SB and have a better understanding of the impacts secondary conditions have on daily life | n=72 adults with SB (25 males, 79% lumbar lesion)  separated into different age groups (n =23 for age range 18–24) recruited from an adult SB clinic  Cross-sectional survey | Spina Bifida Secondary Conditions (SBSC) survey tool - created by the study team | - 69% of the total group perceived presence of mental health problems as a secondary condition  - 53% reported problems with depression  - Several other conditions were also reported, including musculoskeletal and skin related (100%); pain (90%); urological (86%); gastrointestinal (82%); and sleep problems (61%) |
| 31 | Whitney et al., 2019  Mental health disorders and physical risk factors in children with cerebral palsy: a cross‐sectional study  USA | To examine the prevalence of mental health disorders among children with and without CP, and to examine the association between physical risk factors (such as physical activity, sleep duration, pain) and elevated risk of mental health disorders in children with CP | n=111 children with CP (70 males, mean age not reported, median age 12.2, range= 6-17) and n=34161 without CP from the 2016 National Survey of Children's Health (NSCH)  [parent/guardian respondent]  Cross‐sectional study  Note: 59.6% of CP participants and 49.8% of controls were between the ages of 12-17 | Psychiatric diagnosis and NSCH survey design | - Mental health disorders included depression, anxiety, behavior problems, and ADHD  -Children with CP had higher odds of mental health disorders (OR=2.7–7.1, p<0.05) except for ADHD (OR=2.5; 95% CI=0.9–7.1)  - Children with CP have elevated prevalence of mental health disorders (anxiety (30.2%), behavior/conduct problems (27.3%) and multi-morbidity (22.3%) but not depression (7.8%)) even after accounting for physical risk factors  - Rate of ADHD for CP youth was 19.5%  - Low physical activity and pain partially accounts for association between CP and depression |
| 32 | Woodward et al., 2012^a^  Assessing the health, functional characteristics, and health needs of youth attending a non-categorical transition support program  USA | To describe the development, implementation and initial results of an assessment of the health and services needs of youth attending a transition support program | n = 87 youth with special needs  (CP 36%, SB 10%, ASD 6%, Down’s syndrome 17%) (47 males, mean age = 17.5, range= 11-22) recruited from a youth and adult rehabilitation center  Cross-sectional survey | Questionnaire developed by staff with questions adapted from National  Survey of Children with Special Health Care Needs (NS-SCHCN)  National Survey of Children’s Health (NSCH), 2002 National Health Interview Survey [youth and parent response forms] | - 18% of parents reported their youth had an unmet mental health need in the past 12 months  - Youth with special health care needs did not have significantly higher levels of reported need for mental health services than youth in the general population  - Transition to adult life is a crucial step, and increased mental health care needs are often not met |
| 33 | Yang, Chen, Yen & Chen, 2015^b^  Psychiatric diagnoses, emotional-behavioral symptoms and functional outcomes in adolescents born preterm with very low birth weights  Taiwan | To examine the prevalence of emotional and behavioral problems, psychiatric diagnoses and functional outcomes in very low birthweight (VLBW) adolescents | n = 61 adolescents born preterm with VLBW, with CP (24.6%), intellectual disability (21.3%), or ADHD (19.7%) (32 males, mean age = 13.4, range = 12–15) from a medical university data bank  Follow-up survey | Child Behaviour Checklist (CBCL), Chinese version  Wechsler Intelligence Scale Children- IV  Mini International Neuropsychiatric Interview for Children and Adolescents (MINI-KID)  Subject observations, parental report, diagnostic tools | - 17.3 % of the adolescents with VLBW were reported to have emotional or behavioral problems above the clinical cut-off of the CBCL  - Males had higher delinquent, social, attention and total problems than females  - 51% had at least one neuropsychiatric disorder once adolescence was reached among those with VLBW, and 23% had more than one diagnosis  - The three most common psychiatric diagnoses were ADHD (n=12), anxiety (n=2), and ASD (n=2)  - Those with extremely low birth weight (ELBW) had more social problems and more ASD diagnoses in adolescence than those with higher birth weights |

^a^ We included this study based on the following: 46% of the sample had physical disabilities associated with mobility issues (CP, SB); 69% of the sample “needed assistance with personal care such as eating, bathing, dressing, or mobility”; the current emphasis on non-categorical approaches in childhood disability research and illustrated in this study; and, the limited evidence available on the occurrence of mental health problems in this population, all of which encouraged us to be flexible in the inclusion of this paper.

^b^ We included this study based on the following: 24.6% had CP; 32.8% of parents reported their adolescent as having a disabled status (formally issued by the government); individuals born with very low birth rates are at often at risk for mobility restriction and the study reports the results of a specific sub-group (i.e., those with extreme low birth weight); and, the limited evidence available on the occurrence of mental health problems in this population, all of which encouraged us to be flexible in the inclusion of this paper.

1. Mean age and range (or standard deviation) is reported for studies that provided this information [↑](#footnote-ref-1)
2. Only measures related to mental health symptoms/behaviors are included in the table [↑](#footnote-ref-2)
