# Supplementary material for: Mental health problems among adolescents and young adults with childhood-onset physical disabilities: A scoping review
Source: Front Rehabil Sci. 2022 Sep 6;3:904586. doi: 10.3389/fresc.2022.904586 (PMC9485587; doi:10.3389/fresc.2022.904586)
Supplement: Supplementary file 3 [file Table_3_v2.docx]

**Supplementary File 3.** Measures and methods used to capture mental health problems

|  | Measure | Info | N of studies using measure | Studies^a^ |
| --- | --- | --- | --- | --- |
| 1 | Children’s Depression Inventory (CDI) | Parent and youth reports | 7 | 1, 7, 13, 17, 18, 22, 29 |
| 2 | Strengths and Difficulties Questionnaire (SDQ-25) | Parent report | 6 | 2, 5, 21 |
|  |  | Parent & youth forms |  | 6, 19, 20 |
| 3 | Child Behaviour Checklist (CBCL) | Parent report  (social competence subscale for 7) | 5 | 4, 6, 7, 22, 33 |
| 4 | Pediatric Quality of Life Inventory (PedsQL) | Youth & parent report | 3 | 1, 19, 26 |
| 5 | Wechsler Intelligence Scale | To assess IQ | 3 | 2, 6, 33 |
| 6 | Child Attitude Towards Illness Scale (CATIS) |  | 2 | 3, 18 |
| 7 | Child Health Questionnaire (CHQ) | Physical and psychosocial well-being of children independently from the underlying disease  -Pain questions | 2 | 19, 21 |
| 8 | Mental Status Examination |  | 2 | 9, 10 |
| 9 | Mood and Feelings Questionnaire (MFQ) |  | 2 | 11, 26 |
| 10 | Anxiety Checklist for Children and Adolescents |  | 1 | 22 |
| 11 | Autism Diagnostic Observation Schedule (ADOS) |  | 1 | 6 |
| 12 | Behavior Assessment System for Children (BASC-2) | Parent report | 1 | 13 |
| 13 | Behavior Rating Inventory of Executive Function (BRIEF) | Mainly the Metacognition Index scale  Parent report | 1 | 13 |
| 14 | Behavioural Risk Factor Surveillance System (BRFSS) |  | 1 | 23 |
| 15 | Brief Pain Inventory (BPI) | Impact of Pain and Functioning and Well-Being Scale | 1 | 23 |
| 16 | Centre for Epidemiological Studies Depression Scale (CES-D) |  | 1 | 25 |
| 17 | Children’s Attributional Style Questionnaire- Revised (CASQ-R) |  | 1 | 29 |
| 18 | Childhood Health Assessment Questionnaire (CHAQ) | Functional ability in ADLs in children with JIA | 1 | 11 |
| 19 | Children’s Loneliness Scale |  | 1 | 13 |
| 20 | Emotion Regulation Checklist (ERC) | Parent report | 1 | 2 |
| 21 | Family Quality of Life Survey |  | 1 | 14 |
| 22 | Fatigue Assessment Scale (FAS) |  | 1 | 23 |
| 23 | General Health Questionnaire (GHQ- 30) | Parents reporting on their own mental health, in relation to child's mental health | 1 | 19 |
| 24 | Harter’s Rating Scale of Child’s Actual Behaviour | Parent/teacher report | 1 | 7 |
| 25 | Hopkins Symptom Checklist (HSCL-25) |  | 1 | 3 |
| 26 | KIDSCREEN-52 | Parent report | 1 | 21 |
| 27 | Medical Outcome Study short-form Health Survey (SF-36) |  | 1 | 28 |
| 28 | Patient Health Questionnaire-9 (PHQ-9) | Used to measure severity of depression | 1 | 23 |
| 29 | Parenting Stress Index Short Form (PSI) | Parent report | 1 | 21 |
| 30 | Patient Assessment of Chronic Illness Care (PACIC) | Associations with anxiety and depression | 1 | 3 |
| 31 | Revised Children’s Manifest Anxiety Scale (RCMAS-2) |  | 1 | 17 |
| 32 | Reading the Mind in the Eyes Test (RMET) - to assess Theory of Mind (ToM) | Looked at social difficulties | 1 | 2 |
| 33 | Screen for Child Anxiety Related Emotional Disorders Questionnaire (SCARED) |  | 1 | 26 |
| 34 | Social Orientation of Parents with Handicapped Children Questionnaire (SOEBEK) | Measure parent stress (found associations between parental stress and CBCL scores for children) | 1 | 4 |
| 35 | Vineland Adaptive Behaviour Scales - 2 | Parent report, interview | 1 | 5 |
| 36 | Visual Analog Scale | To measure pain | 1 | 11 |

^a.For each study cited within this table, the numbers in the last column represent the selected studies presented (in alphabetical order) in Supplementary File 2: Overview of Studies Reviewed (1-33).^
